# Processed *Aloe vera* Gel Ameliorates Cyclophosphamide-Induced Immunotoxicity

**DOI:** 10.3390/ijms151119342

**Published:** 2014-10-24

**Authors:** Sun-A Im, Ki-Hyang Kim, Hee-Suk Kim, Ki-Hwa Lee, Eunju Shin, Seon-Gil Do, Tae Hyung Jo, Young In Park, Chong-Kil Lee

**Affiliations:** 1 College of Pharmacy, Chungbuk National University, Cheongju 361-763, Korea; E-Mails: littlei@hanmail.net (S.-A.I.); yes3343@hanmail.net (K.-H.K.); dhkslzl@naver.com (H.-S.K.); newghl@nate.com (K.-H.L.); 2 Univera Inc., Seoul 133-120, Korea; E-Mails: ejayshin@univera.com (E.S.); sgildo@univera.com (S.-G.D.); thjo@namyangglobal.com (T.H.J.); 3 College of Pharmacy, Korea University, Sejong 339-700, Korea; E-Mail: yipark@korea.ac.kr

**Keywords:** processed *Aloe vera* gel, polysaccharide, cyclophosphamide, lymphopenia, erythropenia, Peyer’s patch, immunomodulation, IgA

## Abstract

The effects of processed *Aloe vera* gel (PAG) on cyclophosphamide (CP)-induced immunotoxicity were examined in mice. Intraperitoneal injection of CP significantly reduced the total number of lymphocytes and erythrocytes in the blood. Oral administration of PAG quickly restored CP-induced lymphopenia and erythropenia in a dose-dependent manner. The reversal of CP-induced hematotoxicity by PAG was mediated by the functional preservation of Peyer’s patch cells. Peyer’s patch cells isolated from CP-treated mice, which were administered PAG, produced higher levels of T helper 1 cytokines and colony-stimulating factors (CSF) in response to concanavalin A stimulation as compared with those isolated from CP-treated control mice. PAG-derived polysaccharides directly activated Peyer’s patch cells isolated from normal mice to produce cytokines including interleukin (IL)-6, IL-12, interferon-γ, granulocyte-CSF, and granulocyte-macrophage-CSF. The cytokines produced by polysaccharide-stimulated Peyer’s patch cells had potent proliferation-inducing activity on mouse bone marrow cells. In addition, oral administration of PAG restored IgA secretion in the intestine after CP treatment. These results indicated that PAG could be an effective immunomodulator and that it could prevent CP-induced immunotoxic side effects.

## 1. Introduction

The gels of *Aloe* species contain immunomodulatory polysaccharides. Acemannan, which consists of a mixture of polymer chains of β-(1,4)-linked acetylated galactomannan with different lengths, is the best-characterized immunomodulatory polysaccharide [1,2,3]. Acemannan had antitumor activity in spontaneous and transplantation-based animal tumor models [4,5,6,7], antiviral activity in retrovirus-infection models [8,9], and hepatoprotective activity against chronic alcohol-induced liver injury [10]. The antitumor and antiviral activities of acemannan are immune-mediated. The immunomodulatory activities of acemannan are mediated primarily by activation of professional antigen presenting cells (APC) such as macrophages and dendritic cells. Acemannan activated macrophages to produce inflammatory cytokines such as interleukin-1 (IL-1), IL-6, tumor necrosis factor-α (TNF-α) [11], and nitric oxide [12,13], and also upregulated their candidicidal activity [14,15]. In addition, acemannan induced phenotypic and functional maturation of immature dendritic cells [16]. 

The average molecular weight of native polysaccharides in *Aloe vera* gel is 2 MDa or greater [7]. Treatment of viscous *Aloe vera* gel with cellulase not only reduces its viscosity making it easier to process in industrial manufacturing, but it also lowers the average molecular weight of *Aloe* polysaccharides. By using *Aloe* polysaccharides prepared from cellulase-treated *Aloe vera* gel, we previously showed that reducing the molecular size to 5 to 400 KDa enhanced the immunomodulatory activity of the polysaccharides as compared with those isolated from native *Aloe vera* gel [7]. 

Cyclophosphamide (CP) is a widely used antineoplastic drug with broad-spectrum anti-cancer activity [17]. CP is an alkylating agent, which adds an alkyl group to the guanine base of DNA. CP is among the most utilized drugs in chemotherapy, with FDA-approved indications in many different types of cancers including breast cancer, leukemia, Hodgkins and non-Hodgkins lymphoma, multiple myeloma, neuroblastoma, retinoblastoma, and ovarian cancer. However, CP has severe and life-threatening adverse effects. The major toxic side effect of CP is the acute and transient inhibition of hematopoiesis, primarily caused by damage to rapidly proliferating hematopoietic progenitors and their mature progeny leading to leukopenia [18,19,20]. Biopolymers, such as polysaccharides and chitooligosaccharides isolated from natural sources, have been shown to ameliorate CP-induced immunosuppression [21,22,23]. The combined administration of CP with different detoxifying and protective natural products might reduce its toxicity.

In this study, we showed that processed *Aloe vera* gel (PAG) had protective activity against CP-induced immunotoxicity in mice. We demonstrated that oral administration of PAG reversed CP-induced lymphopenia by enhancing the production of hematopoietic cytokines by Peyer’s patch cells. As far as we know, this is the first report on the immunomodulatory effect of *Aloe* polysaccharides on Peyer’s patch cells. Further, we showed that PAG significantly enhanced CP-suppressed IgA secretion in the intestine. 

## 2. Results

### 2.1. Molecular Size Distribution of PAG Polysaccharides

**Figure 1:**
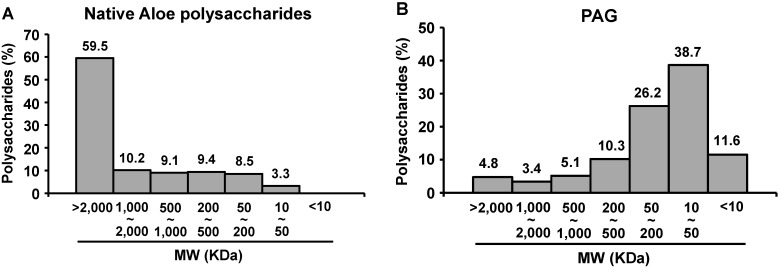
Comparison of the molecular sizes of the polysaccharides in native *Aloe vera* gel (**A**) and in PAG (**B**).

The molecular size distribution of PAG polysaccharides was determined by size exclusion chromatography by using dextran standards. The sizes of PAG polysaccharides were heterogeneous and significantly smaller than those of polysaccharides prepared from native *Aloe vera* gel (Figure 1). Most of the polysaccharides prepared from fresh *Aloe vera* gel were larger than 1 MDa (Figure 1A). In contrast, the majority of PAG polysaccharides were smaller than 500 KDa (Figure 1B). Polysaccharides ranging from 10 to 500 KDa comprised 75.15% of total PAG polysaccharides. The proportion of polysaccharides in the intermediate size range (50–200 KDa) was 26.24% of total PAG polysaccharides. Previously, we showed that polysaccharides between 5 and 400 KDa had potent immunomodulatory activity, while the polysaccharides larger than 400 KDa or smaller than 5 KDa had only marginal immunomodulatory activity [7]. Thus, the PAG used in this study was expected to exert potent immunomodulatory activity. 

### 2.2. Effect of Orally Administered PAG on CP-Induced Lymphopenia and Erythropenia

**Figure 2:**
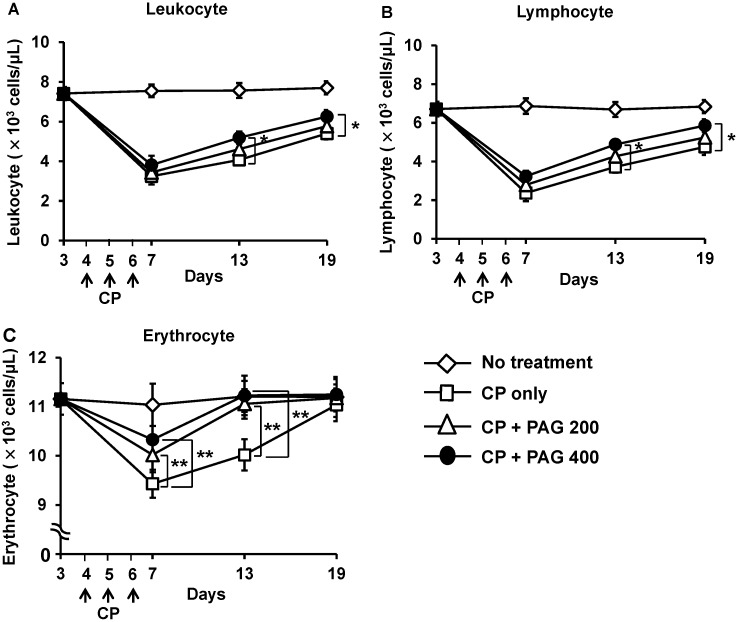
Oral administration of PAG prevents and restores CP-induced lymphopenia and erythropenia. Mice (*n* = 10) were orally administered 200 or 400 mg/kg/day PAG for 19 days. Immunosuppression was induced by intraperitoneal (i.p.) injection of CP (25 mg/kg/day) for three consecutive days starting on the fourth day after PAG administration. The changes in the total numbers of leukocytes (**A**); lymphocytes (**B**); and erythrocytes (**C**) in the blood were examined on the indicated days. * indicates *p* < 0.05 and ** indicates *p* < 0.01.

The changes in the total numbers of leukocytes and erythrocytes in peripheral blood can reflect the toxic side effects of CP. As shown in Figure 2, injection of CP (25 mg/kg/day) for three consecutive days significantly decreased the total numbers of leukocytes and erythrocytes as compared with those in the control group (Figure 2A,C). The CP-induced leukopenia was primarily due to a reduction in total lymphocytes (Figure 2B). The CP-induced lymphopenia and erythropenia were prevented and restored by daily oral administration of PAG. When examined on the next day of final CP-treatment (day 7), the number of erythrocytes in the blood was significantly higher in the PAG-treated group compared with that of the PAG-untreated control group (Figure 2C). The number of lymphocytes was also higher in the PAG-treated group, although the difference was not statistically significant (Figure 1A). Continued daily administration of PAG up to 19 days significantly restored the CP-induced lymphopenia and erythropenia (Figure 1). The restorative activity of PAG on CP-induced hematotoxicity was dose-dependent. Prevention of CP-induced body weight loss could be an indication of the preventive effect of PAG on CP-induced general toxicity. Thus, we measured body weight changes every 2–3 days. However, we were unable to see discernible body weight changes in the CP-treated mice as well as in the CP-treated PAG-treated mice (data not shown). In fact, we had chosen a CP dose (25 mg/kg/day) that did not exert profound toxic side effects while exerting hematotoxicity from prior dose-response experiments. At this dose of CP, there was no noticeable pathological change in the liver and colon. 

### 2.3. Oral Administration of PAG Preserves the Cytokine-Producing Capability of Peyer’s Patch Cells in CP-Treated Mice

**Figure 3:**
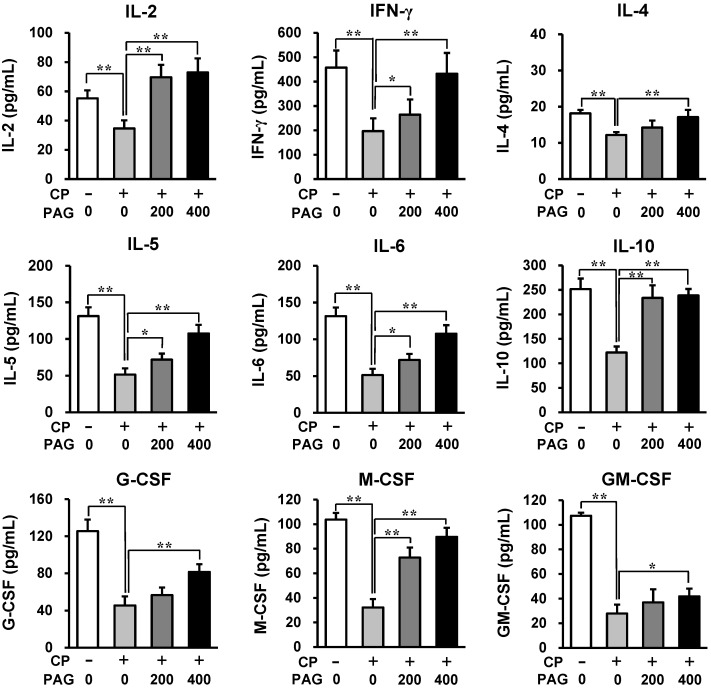
Oral administration of PAG preserves the cytokine-producing capability of Peyer’s patch cells. Peyer’s patch cells were isolated from CP-treated mice that were administered PAG and were then stimulated with Con A for 4 days. Cytokine levels in the cell culture supernatants were measured by using enzyme-linked immunosorbent assays (ELISA). * indicates *p* < 0.05 and ** indicates *p* < 0.01.

To understand the mechanisms underlying the preventive and restorative effect of PAG on CP-induced hematotoxicity, Peyer’s patch cells were isolated from CP-treated mice, which were administered PAG and were then stimulated with 4 μg/mL of concanavalin A (Con A) for 4 days. Con A is a non-specific T cell mitogen; therefore, Con A-stimulated Peyer’s patch T cells are expected to produce numerous cytokines. To analyze the cytokine-producing capability of Peyer’s patch T cells, we focused on the production of T helper 1 (Th1) cytokines (IL-2 and IFN-γ), Th2 cytokines (IL-4 and IL-5), and hematopoietic cytokines (IL-6, M-CSF, G-CSF, and GM-CSF) in response to Con A stimulation. Peyer’s patch cells isolated from CP-treated mice produced much lower levels of these cytokines in response to Con A-stimulation as compared to cells isolated from control mice (Figure 3). In contrast, the Peyer’s patch cells isolated from CP-treated mice that were administered PAG produced significantly higher levels of cytokines (Figure 3). Oral administration of PAG (400 mg/kg) preserved the Th1 and Th2 cytokine-producing capability of Peyer’s patch cells. However, Con A-stimulated Peyer’s patch T cells produced less than 20 pg/mL of IL-4, which is a Th2 cytokine. IL-6, G-CSF, and M-CSF, but not GM-CSF-production, was also almost completely preserved by administration of 400 mg/kg of PAG. PAG administration also increased IL-10-producing capability of Peyer’s patch cells, suggesting that PAG increases the production of regulatory T cell cytokines. These results showed that oral administration of PAG preserved the cytokine-producing capability of Peyer’s patch cells in CP-treated mice.

### 2.4. PAG Polysaccharides Activate Peyer’s Patch Cells to Produce Hematopoietic Cytokines

**Figure 4:**
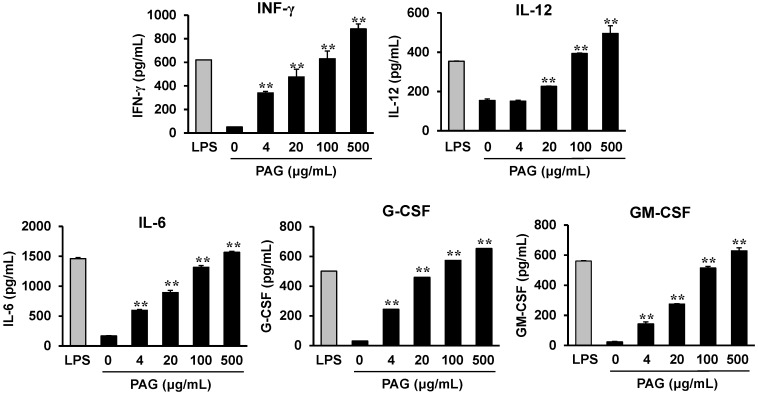
*Aloe* polysaccharides induce hematopoietic cytokine production by Peyer’s patch cells. Peyer’s patch cells isolated from control mice were stimulated with *Aloe* polysaccharides for 3 days. The cytokine levels in the culture supernatants were determined by using ELISA. ** indicates *p* < 0.01, compared to PAG-untreated group.

To determine the effect of PAG polysaccharides on hematopoietic cytokine production by Peyer’s patch cells, PAG polysaccharides, which were purified by extensive dialysis by using molecular weight cut-off membranes (MWCO 3500) to remove small MW substances, were added to cultures of Peyer’s patch cells isolated from normal mice. After 3 days, the cell culture supernatants were collected and the levels of Th1-associated cytokines (IFN-γ and IL-12) and hematopoietic cytokines (IL-6, G-CSF, and GM-CSF) were analyzed. The polysaccharides induced cytokine production by Peyer’s patch cells in a dose-dependent manner (Figure 4). These results showed that the polysaccharides directly activated Peyer’s patch cells to produce different cytokines. 

### 2.5. Cytokines Produced by Polysaccharide-Stimulated Peyer’s Patch Cells Promote Bone Marrow Cell Proliferation 

**Figure 5:**
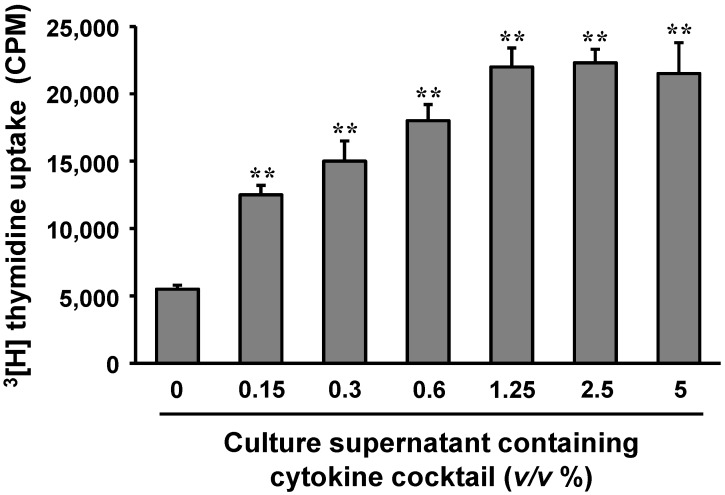
Effects of cytokines produced by Peyer’s patch cells stimulated with the *Aloe* polysaccharides on the proliferation of bone marrow cells. Peyer’s patch cells isolated from control mice were stimulated with 100 μg/mL of polysaccharides for 3 days. The indicated amounts of the cytokine-containing cell culture supernatants (*v*/*v* %) were added to cultures of mouse bone marrow cells. ** indicates *p* < 0.01, compared to culture supernatant-untreated group.

The effect of cytokines produced by polysaccharide-stimulated Peyer’s patch cells on hematopoiesis was examined by using mouse bone marrow cells. Peyer’s patch cells isolated from control mice were cultured in the presence of 100 μg/mL polysaccharides for 3 days. The cell culture supernatant was collected and added to the culture medium of mouse bone marrow cells. As shown in Figure 5, the Peyer’s patch cell culture supernatant containing hematopoietic cytokines induced the proliferation of mouse bone marrow cells in a dose-dependent manner. 

### 2.6. Oral Administration of PAG Restores CP-Suppressed Immunoglobulin A (IgA) Secretion in the Intestine 

**Figure 6:**
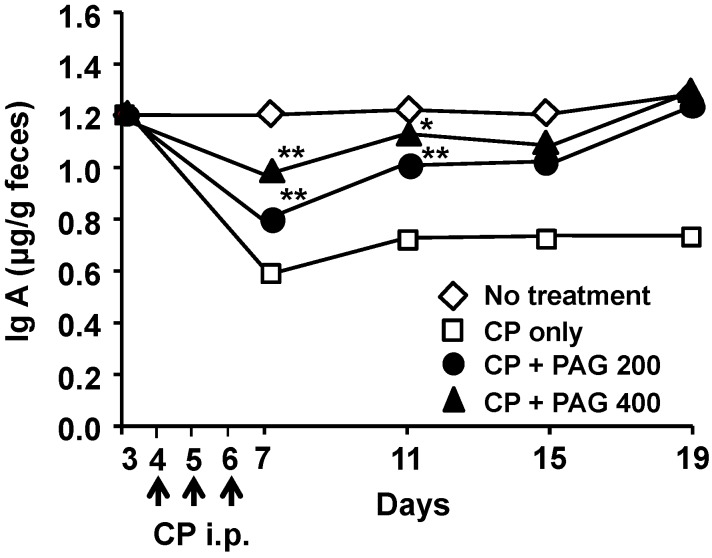
Effect of oral administration of PAG on CP-suppressed IgA production in the intestine. Fresh feces from mice from each group were collected on days 3, 7, 11, 15, and 19. The feces were freeze-dried, weighed, and dissolved in phosphate-buffered saline containing protease inhibitors. Total IgA levels in fecal extracts were assessed by using an ELISA. * indicates *p* < 0.05 and ** indicates *p* < 0.01.

IgA plays an important role in host defense in the mucosal immune system. Peyer’s patches are known as inductive sites for IgA production. Thus, we examined the effects of oral administration PAG on intestinal IgA secretion in CP-treated mice. Fresh feces from mice from each group were collected on days 3, 7, 11, 15, and 19 and the IgA levels in the fecal extracts were measured. As shown in Figure 6, injection of CP (25 mg/kg/day) for three consecutive days significantly decreased IgA levels in the feces. One day after the third injection of CP, the fecal IgA content was reduced to approximately 50% as compared with that in the control group. The CP-suppressed intestinal IgA secretion was prevented and restored by daily oral administration of PAG (Figure 6). When examined on the next day of final CP-treatment, the fecal IgA content was significantly higher in the PAG-treated group compared with PAG-untreated control group. Continued daily administration of PAG up to 19 days almost completely restored the CP-suppressed IgA secretion to the normal control level.

## 3. Discussion 

In this study, we showed that oral administration of PAG ameliorated CP-induced immunotoxicity in mice. Lymphopenia and erythropenia are the major toxic side effects of CP [17,18,19,20]. PAG prevented and restored CP-induced erythropenia. PAG also demonstrated moderate preventive effect for CP-induced lymphopenia with a better restoration effect in dose correlation. PAG administration also prevented and restored CP-suppressed IgA secretion in the intestine. We further found that PAG protected Peyer’s patch cells from CP-induced damage, which could be the underlying mechanism for the protective activity of PAG on CP-induced immunotoxicity. In addition, we showed that the PAG polysaccharides directly activated Peyer’s patch cells to produce different cytokines including IL-6, IL-12, IFN-γ, G-CSF, and GM-CSF.

Peyer’s patches are one of the most important components of gut-associated lymphatic tissues and they play an essential role in initiating IgA production. Peyer’s patches are distributed throughout the intestinal wall and are separated from the intestinal lumen by a single layer of epithelium containing microfold cells (M cells), which are able to engulf antigens from the lumen and deliver them to APCs [24,25]. These tissues are infiltrated by a large number of T-cells, B-cells, macrophages, and dendritic cells. When pathogenic antigens enter through the M-cells, they are captured by APCs for presentation to naive T-cells [26,27]. Following their interaction with APCs in Peyer’s patches, the mature lymphocytes migrate further to reach the bloodstream. Therefore, Peyer’s patches play important roles in the regulation of the mucosal and systemic immune systems [28]. 

Our study showed that PAG protected the functional activity of Peyer’s patch cells from CP-induced damage. We found that the cytokine-producing capability of Peyer’s patch cells isolated from CP-treated mice was significantly suppressed as compared to that of cells isolated from control mice. The CP-induced suppression of cytokine production was almost completely reversed by oral administration of PAG. The protective effects of PAG on the cytokine-producing capability of Peyer’s patch cells after CP treatment were evident on Th1 cytokines (IL-2 and IFN-γ) and hematopoietic cytokines (IL-6, G-CSF, and M-CSF). IL-2 and IFN-γ are the major cytokines produced by naive T cells upon activation by professional APCs such as macrophages and dendritic cells [29]. IL-6 is a pleiotropic cytokine that regulates multiple biological processes, including the development of the nervous and hematopoietic systems, acute-phase responses, inflammation, and immune responses [29]. IL-6 was shown to be required for M-CSF and G-CSF activity during hematopoiesis [30,31,32,33]. In this study, we showed that PAG polysaccharides directly activated Peyer’s patch cells to produce Th1-associated cytokines (IL-12 and IFN-γ) and hematopoietic cytokines (IL-6, G-CSF, and M-CSF), which was sufficient to induce proliferation of mouse bone marrow cells. IL-12 stimulated Th0 cells to differentiate into Th1 cells [34]. It should be noted that an increase in lymphocyte proliferation and activation is not always beneficial because non-specific activation of the immune system could result in organ damage or autoimmunity.

The major immunomodulatory polysaccharide in PAG is acemannan [7,16]. Acemannan was shown to exert antitumor [4,5,6,7] and antiviral activity [8,9]. The antitumor and antiviral activities of acemannan are immune-mediated, and it targets primarily professional APCs such as macrophages and dendritic cells [11,12,13,14,15,16]. Thus, the protective activity of PAG on CP-induced Th1 cell immunotoxicity could be mediated via cytokines and co-stimulatory molecules produced by acemannan-activated professional APCs. 

IgA is produced in the intestinal epithelium and secreted into the lumen thereby providing protection against microbes, neutralizing pathogenic bacteria, and controlling commensals. The secreted IgA found in the intestinal lumen is produced by plasma cells that migrate from Peyer’s patches or other mucosa-associated lymphoid tissues in response to epithelial signals [35]. As shown in Figure 6, treatment with CP (25 mg/kg/day) for three consecutive days significantly decreased the IgA content in the feces. Oral administration of PAG restored the fecal IgA level to normal in a dose-dependent manner. The mechanism through which PAG induces IgA production remains unclear. 

## 4. Experimental Section 

### 4.1. Preparation and Molecular Weight Determination of PAG

PAG was prepared from the gel of *Aloe vera*, as described previously [7,36]. The basic methodology used to prepare PAG involved incubation of the *Aloe vera* gel with cellulase, termination of the reaction by heating, and filtration through a charcoal column to remove anthraquinones and other colored substances. The molecular weight of the polysaccharides in the PAG was determined by comparing their retention time with that of broad dextran standards (Phenomenex Co., Torrance, CA, USA) by using a TSKgel G4000 PWxl column (7.8 mm × 30 cm; Tosoh Bioscience, Shiba, Japan) as described earlier [7]. Polysaccharides or dextran standards in the column were eluted with distilled water, and were then detected with an ELSD2000 detector (Alltech Associates Co., Deerfield, Illinois, IL, USA). In some experiments, the PAG polysaccharides were further purified by extensive dialysis against distilled water by using a molecular weight cut-off membrane (MWCO 3500; Spectrum Laboratories, Inc., Rancho Dominguez, CA, USA).

### 4.2. Animals, Immunosuppression, and Treatment Protocol

Six-week-old C57BL/6 female mice were purchased from OrientBio Inc. (Seongnam, Korea), and C3H/HeJ mice were purchased from SLC Inc. (Shizuoka, Japan). The mice were housed five per cage under specific pathogen-free conditions and were treated according to the institutional protocols approved by the Animal Care Committee of Chungbuk National University. Mice were randomly divided into four groups. One group of healthy mice was used as a control. The other three groups of mice were treated with CP (25 mg/kg/day) by i.p. injection for three consecutive days to induce immunosuppression. To examine the effects of PAG, mice were orally administered PAG at doses of 40 or 80 mg/kg/day for consecutive 19 days starting three days before the first CP injection. 

### 4.3. Blood Analysis

Blood samples were collected by submandibular vein puncture and immediately transferred into tubes containing K_2_-EDTA (BD, Franklin Lakes, NJ, USA) on days 3, 7, 13, and 19 after the first day of PAG administration. Blood samples were used to determine complete blood cell counts by using an Abbot CellDyn-3500 automated hematology analyzer (Abbott Laboratories, Chicago, IL, USA).

### 4.4. Peyer’s Patch Cell Preparation and Culture

After sacrificing the mice, small intestines were excised, placed in a Petri dish, and washed extensively with ice-cold phosphate-buffered saline (PBS) containing 100 U/mL penicillin (Invitrogen, Carlsbad, CA, USA) and 100 μg/mL streptomycin (Invitrogen). Visible Peyer’s patches were carefully isolated from the wall of the small intestine by using fine scissors. The isolated Peyer’s patches from each mouse were pooled together and washed twice with ice-cold PBS, and single cell suspensions were obtained by straining through a 70-µm cell strainer (BD Bioscience). The cell viability was over 95% as determined by using the Trypan blue exclusion method. The cells were cultured in Dulbecco’s modified Eagle’s medium (Hyclone Laboratories Inc., Logan, Utah, UT, USA) supplemented with 10% heat-inactivated fetal bovine serum (Hyclone), 100 U/mL penicillin and 100 µg/mL streptomycin (Invitrogen), and 50 µM 2-mercaptoethanol (Sigma-Aldrich, St. Louis, MO, USA) at 37 °C in a humidified atmosphere containing 5% CO_2_.

### 4.5. Cytokine Production and Measurement

Peyer’s patch cells were cultured for four days in the presence or absence of concanavalin A (Con A; Sigma-Aldrich) in a 24-well plate at 3 × 10^6^ cells/well in a total volume of 1 mL. After 4 days, the cell culture supernatants were collected and the levels of different cytokines were measured by using ELISA kits (BD Biosciences, San Diego, CA, USA) according to the manufacturer’s instructions.

### 4.6. Measurement of IgA Content in the Feces 

Fresh feces from mice from each group were collected on days 3, 7, 11, 15, and 19. The feces were freeze-dried, mixed in PBS containing 1 mM phenylmethylsulfonyl fluoride and 0.3 μM aprotinin for 2 h at 4 °C, and centrifuged at 12,000 rpm for 10 min. The resulting supernatant was used to determine IgA levels in fecal extracts. Total IgA levels in fecal extracts were assessed by using an ELISA in which the anti-mouse IgA capture antibody (clone C10.3; Pharmingen, San Diego, CA, USA) was coated on the plate and it was detected with a biotinylated anti-mouse IgA antibody (clone C10.1). IgA content was expressed as μg of IgA/g feces.

### 4.7. Statistical Analysis

Student’s *t*-test was performed for a single comparison of two groups after an evaluation for normality. A Mann-Whitney rank sum test was performed if the data distribution was not normal. One- or two-way analysis of variance was applied for comparisons of multiple groups.

## 5. Conclusions

Oral administration of PAG prevented and restored CP-induced lymphopenia and erythropenia in a dose-dependent manner. The protective activity of PAG on CP-induced immunotoxicity is mediated by the functional preservation of Peyer’s patch cells from CP-induced damage. The active component in the PAG is *Aloe* polysaccharides. PAG administration also prevented and restored CP-suppressed IgA secretion in the intestine.
